# CD13 facilitates immune cell migration and aggravates acute injury but promotes chronic post-stroke recovery

**DOI:** 10.1186/s12974-023-02918-3

**Published:** 2023-10-10

**Authors:** Justin N. Nguyen, Eric C. Mohan, Gargee Pandya, Uzma Ali, Chunfeng Tan, Julia K. Kofler, Linda Shapiro, Sean P. Marrelli, Anjali Chauhan

**Affiliations:** 1 University of Texas McGovern Medical School at Houston, Houston, TX USA; 2 Department of Neurology, University of Texas McGovern Medical School at Houston, Houston, TX USA; 3 Baylor University, Waco, TX USA; 4 Department of Pathology, University of Pittsburgh, Pittsburgh, PA USA; 5 Center for Vascular Biology, The University of Connecticut Health Center, Farmington, CT USA

**Keywords:** CD13, Ischemic stroke, Stroke recovery, Cognitive impairment, CXCL12, CXCR4, Angiogenesis, Myeloid cells, Transmigration

## Abstract

**Introduction:**

Acute stroke leads to the activation of myeloid cells. These cells express adhesion molecules and transmigrate to the brain, thereby aggravating injury. Chronically after stroke, repair processes, including angiogenesis, are activated and enhance post-stroke recovery. Activated myeloid cells express CD13, which facilitates their migration into the site of injury. However, angiogenic blood vessels which play a role in recovery also express CD13. Overall, the specific contribution of CD13 to acute and chronic stroke outcomes is unknown.

**Methods:**

CD13 expression was estimated in both mice and humans after the ischemic stroke. Young (8–12 weeks) male wild-type and global CD13 knockout (KO) mice were used for this study. Mice underwent 60 min of middle cerebral artery occlusion (MCAO) followed by reperfusion. For acute studies, the mice were euthanized at either 24- or 72 h post-stroke. For chronic studies, the Y-maze, Barnes maze, and the open field were performed on day 7 and day 28 post-stroke. Mice were euthanized at day 30 post-stroke and the brains were collected for assessment of inflammation, white matter injury, tissue loss, and angiogenesis. Flow cytometry was performed on days 3 and 7 post-stroke to quantify infiltrated monocytes and neutrophils and CXCL12/CXCR4 signaling.

**Results:**

Brain CD13 expression and infiltrated CD13^+^ monocytes and neutrophils increased acutely after the stroke. The brain CD13^+^lectin^+^ blood vessels increased on day 15 after the stroke. Similarly, an increase in the percentage area CD13 was observed in human stroke patients at the subacute time after stroke. Deletion of CD13 resulted in reduced infarct volume and improved neurological recovery after acute stroke. However, CD13KO mice had significantly worse memory deficits, amplified gliosis, and white matter damage compared to wild-type animals at chronic time points. CD13-deficient mice had an increased percentage of CXCL12^+^cells but a reduced percentage of CXCR4^+^cells and decreased angiogenesis at day 30 post-stroke.

**Conclusions:**

CD13 is involved in the trans-migration of monocytes and neutrophils after stroke, and acutely, led to decreased infarct size and improved behavioral outcomes. However, loss of CD13 led to reductions in post-stroke angiogenesis by reducing CXCL12/CXCR4 signaling.

**Supplementary Information:**

The online version contains supplementary material available at 10.1186/s12974-023-02918-3.

## Introduction

Worldwide, stroke is the third leading cause of death [1]. Injury progression involves both acute (within minutes to hours) and chronic mechanisms [2]. During the acute phase of stroke, peripheral immune cells, including monocytes and neutrophils, infiltrate the brain and lead to neuroinflammation [3]. However, restorative processes including angiogenesis and neurogenesis are stimulated in the later stages that are conducive to stroke recovery [2]. Recovery starts days to weeks after ischemic stroke [4]. Angiogenesis is detected in the peri-infarct area in both animals and humans [5, 6] and is positively correlated with survival and stroke outcomes [7–11]. Activated endothelial cells express adhesion molecules and recruit inflammatory cells [12–14]. These inflammatory cells then produce soluble factors, such as chemokines and cytokines that influence endothelial cell function and promote angiogenesis [12–14]. Thus, the development of efficacious drugs will depend on the differential targeting of both early inflammatory responses and the delayed reparative processes after stroke.

Stroke induces profound time-dependent systemic effects, including the activation of circulating leukocytes [15]. Monocytes and neutrophils are stimulated within minutes of ischemic injury [15]. These cells express adhesion molecules and invade the brain leading to inflammation [16]. CD13 is a metalloprotease, expressed on myeloid cells, pericytes, fibroblasts, epithelial and endothelial cells, as well as on tumor cells, and stem cells [17–22]. CD13 acts as an adhesion molecule that modulates inflammatory immune cell trafficking resulting in the progression of injury [23–25]. Activation and crosslinking of reciprocal CD13 molecules in monocytes and endothelium results in monocyte trafficking to the site of injury [24, 26]. Furthermore, CD13 receptors on activated neutrophils interact in a homotypic fashion [27], which could lead to occlusive plugs causing tissue hypoxia and endothelial damage. Once across the blood–brain barrier (BBB), infiltrating immune cells release pro-inflammatory cytokines and activate proteases, leading to the amplification of brain inflammatory responses and can result in disruption of the BBB, neuronal cell death, and hemorrhagic transformation [28, 29]. Within the vasculature, CD13 is exclusively expressed by angiogenic/activated endothelial cells [30, 31]. Angiogenic blood vessels in the peripheral circulation express CD13 after ischemia in both skeletal muscle and the heart and play an important role in subsequent repair [23, 32]. These findings suggest that CD13, in addition to its role in inflammation, may also play a role in promoting recovery. We hypothesized that CD13 contributes to increased myeloid trans-migration and worsens outcomes in the acute phase of stroke, whereas it contributes to reparative angiogenesis and improved outcomes in the chronic phase of stroke. We tested this hypothesis using global CD13 knockout mice examined at acute and chronic phases of stroke. Our findings revealed that knockout of CD13 resulted in reduced brain infiltration of neutrophils, brain infarct volume, and neurological deficits in the acute phase. In contrast, knockout of CD13 demonstrated reduced angiogenesis and increased cognitive impairment in the chronic phase. Together, these data suggest that targeting CD13 function may be beneficial in the acute phase of stroke, but detrimental continued into the chronic phase.

## Materials and methods

### Animals

Wild type (WT) and CD13 knockout mice (KO) on the C57BL/6 background were a gift from Dr. Shapiro at UConn Health, Farmington, CT, USA. Young adult male mice (8–12 weeks) were used for this study and were group housed in the pathogen-free facility and had access to food and water ad libitum. All animals were group housed in Tecniplast individually ventilated cage racks, fed a commercially available irradiated, balanced mouse diet (no. 5058, LabDiet, St Louis, MO), and provided corncob bedding. Rooms were maintained at 21-24 °C and under a 12:12 h light: dark cycle. Animal procedures were performed at an Association for Assessment and Accreditation of Laboratory Animal Care (AAALAC) accredited facility and were approved by the Animal Welfare Committee at the University of Texas Health Science Center in Houston, TX, USA. All the surgeries and behavioral testing were conducted between 6 and 9 am by an investigator blinded to genotype. All human tissue samples were obtained from the University of Pittsburgh neurodegenerative brain bank with appropriate ethics committee approval (Committee for Oversight of Research and Clinical Training Involving Decedents). All brain sections were from the mid-frontal cortex from the middle cerebral artery territory as determined by expert neuropathological assessments of H&E-stained sections. CD13 brain IHC—the sections were from [controls; 68–90 years, *n* = 9, females (*n* = 3) and males (*n* = 6)], acute ischemic stroke (AIS); 67–89 years, *n* = 8, females (*n* = 4) and males (*n* = 4)]. For CXCL12 brain IHC—[controls; 61–92 years, *n* = 8, females (*n* = 2), males (*n* = 6), AIS; 67–89 years, *n* = 9, females (*n* = 5) and males (*n* = 4)].

### Middle cerebral artery occlusion (MCAO)

Transient focal ischemia was induced under isoflurane anesthesia in the young for 60 min by occlusion of the right middle cerebral artery [3]. Body temperature was maintained at 37.0 ± 1.0 °C throughout the surgery by an automated temperature control feedback system (TC1000, mouse, CWE Inc., USA). A midline ventral neck incision was made, and unilateral MCAO was performed by inserting a Doccol monofilament (Doccol Corp, Redlands, CA, USA) through a right external carotid artery into the internal carotid artery. Cerebral blood flow (CBF) was measured by a Laser Doppler flowmeter (Moor Instruments Ltd., Devor, UK) to ensure occlusion (> 80 percent from baseline) and later MCA recanalization. Animals were allowed to awaken from anesthesia during the intra-ischemic period to ensure behavioral deficits (turning). One hour after ischemia, animals were re-anesthetized, and reperfusion was established by the withdrawal of the monofilament. Animals were then placed in a recovery cage and were euthanized 72 h after reperfusion (for acute phase studies). For chronic studies, mice were euthanized at day 30 post-MCAO. Ipsilateral cortical perfusion was evaluated by Laser speckle perfusion imaging using MoorFLPI full-field laser perfusion imager (Moor Instruments, Devon, UK) at day 30 post-MCAO. Sham controls underwent the same procedure except the monofilament was not introduced to occlude the middle cerebral artery. Animals were randomly assigned to stroke and sham surgery groups and housed in recovery cages for 2 h after surgery. Sham and stroke mice were then housed together in their home cages (group housing) to minimize the detrimental effects of social isolation [33]. Mice used for stroke (or sham) underwent pre-screening with the Barnes maze. All mice escaped to the dark box in the Barnes maze within 3 min and were thus included in the study. Two mice died in WTMCAO group (days 5 and 10 post-MCAO), and one mouse died in the CD13KO MCAO group (day 5). These mice were excluded from the day 7 analysis, but were included in the pre-MCAO analysis. BrdU (Sigma, 50 mg/kg, i.p.) was administered for 10 days starting on day 1 post-MCAO.

### Behavioral testing

Neurological deficit scoring was done on day 3 post-MCAO and the mice were euthanized after the testing. Open field, Y-maze, and Barnes maze were performed on day 7 and day 28 post-MCAO in the chronic cohort. The animals were euthanized at day 30 post-MCAO. Behavioral studies were analyzed by a trained observer blinded to surgical group and genotype. The testing apparatus was cleaned with 70% ethanol between mice.

### Neurological deficit scores

The NDS was assessed on day 3 post-MCAO to quantify acute neurological deficits [34]. The standard scoring system was as follows: 0, no deficit; 1, forelimb weakness and torso turning to the ipsilateral side when held by the tail; 2, circling to affected side; 3, unable to bear weight on affected side; and 4, no spontaneous locomotor activity or barrel rolling.

### Open field-testing

Locomotor activity was assessed as described previously on days 7 and 28 after MCAO [35]. Mice were placed in a brightly lit box, 50 cm wide × 50 cm length × 38 height, and allowed to explore freely for 5 min. The mice were filmed from above throughout the test. These videos were then analyzed using Noldus Ethovision behavior software (Leesburg, VA).

### Y-maze

The Y-maze (90 cm long × 90 cm wide x 76 cm high) apparatus consists of three arms in the shape of a Y in a protocol adapted from Kraeuter et al., 2019 [36]. Mice were placed in the center of the device where the three arms meet. Each respective arm was blocked by a removable barrier. Mice remained in the center for one minute to allow acclimatization to the apparatus. Following this, all barriers were removed simultaneously and mice were allowed to explore the maze for 5 min. Each session was also recorded using a digital video camera (JVC Everio, Victor Company, Japan). Sequences were divided into three groups: spontaneous (e.g., ABC, CBA, CAB, etc.), same arm return (AA, BB, and CC), and alternate arm return (e.g., CAC, BAB, ABA, etc.). The percentage of spontaneous alternations was calculated as [(number of alterations)/(total arm entries − 2)] × 100.

### Barnes maze

The Barnes maze was performed on the elevated circular platform (diameter-9 cm) with 20 equally spaced holes (diameter-5 cm). A randomly chosen hole was designated as the escape hole that allowed the mouse to escape the platform in a dark box below. Training phase (before the surgery): the mouse was placed at the center of the maze and allowed to explore for 5 min. The surrounding walls had visual cues for orientation to the position of the escape box. At the end of 5 min, the mouse was gently moved to the dark box and remained there for 1 min. On days 2–4, the mouse was allowed to explore the maze for 3 min, and the time to escape and the number of incorrect entries was recorded. If the mouse failed to locate the dark box within 3 min, it was gently guided toward the box. Mice received the training twice a day followed by testing on day 5. On day 5 (baseline testing)—the mice performed the task twice and the average was taken. On days 2–5 (training and baseline), 7, and 28 (testing days), peanut butter was placed in the dark box as an olfactory cue to motivate mice to locate the dark box. Mice that located the dark box within the 3 min were enrolled in the study. All the mice that performed the task were able to locate the dark box within 3 min. The mice were then tested on days 7 and 28 post-MCAO to assess long-term memory retention.

### Nest building score

The nest building test was performed and analyzed as described previously [37, 38]***.*** Mice were singly housed overnight in a cage on corncob bedding with one square Nestlet (2X2 inches). Twelve hours later pictures were taken of the nests and scored using a 5-point scale. 1. The nestlet is untouched with over 90% still intact. 2. The nestlet is partially torn up, but over 50% is still intact. 3. The nestlet is almost or completely torn up, but no identifiable nest site is present (pieces scattered throughout the cage). 4. The nestlet is almost or completely torn up and there is an identifiable nest site but the sides of the nest are flat on more than 50% of its circumference. 5. The nestlet is almost or completely torn up and there is an identifiable nest site where the walls of the nest are higher than the body of the mouse on all sides.

### Tissue harvesting

Mice were euthanized on day 3 (acute cohort) and day 30 (chronic/recovery cohort) post-MCAO. Animals were transcardially perfused with 40 mL of cold sterile PBS. The olfactory bulb, brainstem, and cerebellum were removed.

### TTC staining

Brains were placed at − 80 °C for 4 min to slightly harden the tissue. Five, 2 mm coronal sections were then cut from the frontal pole to the cerebellar junction and stained with 1.5% TTC (SIGMA, St. Louise, MO). Slices were formalin-fixed (4%) and then digitalized for assessing infarct volume using Fiji software (release 2.9.0). Infarct volume, mm^3^ = infarct- (contra/Ipsi).

### Cresyl violet (CV) staining

CV staining was performed as described previously [38]. The brain was post-fixed overnight in 4% PFA and placed in 30% sucrose solution for 48 h before processing. The brains were then cut into 30-μm sections on a freezing microtome and every eighth slice was stained by CV to visualize tissue loss. The slices were digitally imaged, and tissue atrophy was analyzed using computer software (Fiji software). Tissue atrophy percentage was calculated by using the following formula: percentage tissue atrophy = (total ipsilateral tissue/total contralateral tissue) × 100.

### Ventricle size area analysis

Three coronal sections were taken from the CV images representing positions of + 0.48, + 0.72, and + 1.92 mm from bregma. The ventricle was identified and the area from the contralateral and ipsilateral mouse brain was quantified on Fiji software. The ventricle area was normalized to the contralateral ventricle. The analysis was repeated twice to ensure the reproducibility of the results.

### Western blot

The ipsilateral cortical tissue was homogenized using lysis buffer (1%NP-40, 1 mM phenylmethylsulfonyl fluoride, protease inhibitors; complete and miniphosStop tablets). Protein concentration was confirmed by BCA Protein Assay Kit (Thermo Fisher Scientific Inc, Rockford IL). The brain lysate was dissolved in a 2% loading buffer and resolved on 4–20% gradient SDS –PAGE and transferred to a polyvinylidene difluoride membrane. Blots were blocked with 5% bovine albumin serum for an hour and incubated with an anti-CD13 antibody (abcam#ab227663; 1:1000) overnight at 4 °C. The CD13KO ipsilateral cortical tissue was run as a control to validate the antibody (Additional file 1: Fig. S1C). The next day, the blots were washed and incubated with an HRP-conjugated secondary antibody (1:10,000) for an hour followed by the detection of the signal by an ECL detection kit. Band intensities were quantified using Fiji software.

### Leukocyte infiltration

The brain was harvested at 72 h post-MCAO and placed in RPMI (Lonza) medium and mechanically and enzymatically digested in collagenase/dispase (1 mg/mL) and DNAse (10 mg/mL; Roche Diagnostics) as described previously [3]. The cell suspension was filtered through a 70-µm filter. Leukocytes were harvested from the interphase of a 70%/30% Percoll gradient. Brain leukocytes were then washed with 1X PBS and blocked with mouse Fc Block (eBioscience, 1 μl/50 μl) before staining with primary antibody-conjugated fluorophores (CD45-ef450, CD11b- APC-ef780, Ly6C-APC, Ly6G- FITC) for 30 min. The infiltrated inflammatory monocytes were identified as CD45^hi^CD11b^+^Ly6C^hi^ and neutrophils were identified as CD45^hi^CD11b^+^Ly6G^+^. For live/dead discrimination, cells were treated with a fixable viability dye (carboxylic acid succinimidyl ester, AF350; Invitrogen).

### Quantification of CD13^+^ endothelial or mural cells

The brains were harvested at 24, 72 h, and 7 days post-MCAO as described previously with modifications [39]. The brains were placed in an RPMI medium and mechanically digested. The cell suspension was filtered through a 70-µm filter and centrifuged at 300 g for 5 min at 4 °C. The pellet was mixed with 4 ml of 22% bovine serum albumin (BSA) and centrifuged at 300 g for 20 min at 4 °C (acceleration -4 and deceleration -0). Myelin on top was carefully removed and the cell pellet was washed with RPMI medium and blocked with mouse Fc Block and primary antibody-conjugated fluorophores (CD45-ef450, CD11b-APC-ef780, CD13-BV605, CD146-PE/Cy7, CD31-BV605, CXCR4-APC). For intracellular CXCL12 staining, the cells were permeabilized and fixed by using a fixation/permeabilization kit (BD Biosciences) according to the manufacturer’s instructions. The cells were then incubated with CXCL12-PE conjugated antibody overnight at 4 °C and then washed. Data were acquired on CytoFLEX cytometer (BECKMAN COULTER) and analyzed by FlowJo (TREESTAR INC.). Cell-specific fluorescence minus one control was used to confirm individual antibody specificity. For CD13 antibody specificity, CD13KO mouse brain was used. No less than 300,000 events were recorded for leukocyte estimation. For estimations of CD13^+^ cell counts in the brain no less than 200,000 events were recorded. For estimating CXCL12 and CXCR4^+^ ECs, no less than 1 × 10^6^ events were recorded/sample.

### Immunohistochemistry (IHC)

For the mouse brain, a standard procedure was utilized for IHC staining on 30-μm sections mounted on Fisher Scientific Superfrost Plus charged slides [38]. Briefly, the tissue sections were rinsed in 0.1 M phosphate-buffered saline (PBS) pH 7.4. Antigen retrieval was performed by heating the tissue in a 10 mM sodium citrate buffer at pH 6.0. Tissue sections were incubated for 1 h in blocking solution (0.1% Triton-X, 10% normal goat serum in 1X PBS) and then incubated overnight in the primary antibody at 4 °C (CD13-Alexa Fluor 647 (BD Biosciences#564352); 1:200; IBA-1(Fujifilm Wako pure chemical corporation#NCNP24) 1:300; GFAP-Cy3 (Sigma-Aldrich#C9205) 1:300; DyLight 594 labeled Lycopersicon Esculentum (tomato) lectin (Vector Laboratories); 1:100, MBP (cell signaling technologies#78896S); 1:100, PDGFR-β (Santa Cruz#sc-374573); 1:300, NeuN-Alexa Flour 488 (Millipore Sigma MAB377X); 1:300. Digital images were taken on Lieca microscope. Three coronal brain sections per mouse, taken 0.02, 0.45, and 0.98 mm from bregma, were stained and visualized for quantification at 20× magnification at the core/penumbra junction to quantify positive cells. To assess the number of GFAP and Iba-1 positive astrocytes and microglia in the hippocampus sections from bregma -1.46 mm to bregma-2.30 mm were used. Three images were taken from each mouse brain and values were averaged.

For the human brain, a standard procedure was utilized for IHC staining as described previously [40]. Briefly, after deparaffinization, the sections were subjected to a heat-induced antigen retrieval process (citric acid buffer, pH 6.0, 99 °C 20 min), then blocked in 3% H2O2 to block the endogenous peroxidase. ABC-HRP kit (VECTASTAIN Elite ABC-HRP kit, peroxidase) was used to perform the following process. Briefly, the sections were blocked in blocking buffer (1.5% horse normal serum in PBS), then incubated overnight with primary antibodies, Anti-CD13 (1:200, Abcam, ab227663) and anti-CXCL12 (1:50, R&D Systems, MAB350-100). Samples were washed and subsequently incubated with a secondary antibody (biotin-labeled horse anti-mouse IgG) and avidin–biotin complex. Diaminobenzidine chromogen was used to detect the immunoperoxidase signal, and hematoxylin was used as the counterstain. Negative controls were performed by omitting primary antibodies from the procedure. Digital images were obtained with Leica THUNDER Imager DMi8, using the 20×X objective. Five images/cases were taken, which included infarct area in stroke cases and normal cortical region in control cases. The infarct regions were identified in H&E staining.

### Quantification of angiogenesis

Following three washes with 0.1 M PBS, pH 7.4 for 10 min, sections were placed in ice-cold 1N hydrochloric acid (HCl) for 10 min, followed by incubation with 2N HCl for 30 min at 37 °C. After 30 min, the sections were rinsed three times with 0.1 M 1× PBS and then sections were incubated with 0.1 M boric acid for 10 min. The sections were washed three times and blocked with blocking solution and incubated with BrdU antibody (Santa Cruz biotechnology, sc-32323 AF594) and with *Lycopersicon esculentum* (tomato) lectin, DyLight 649 for 48 h at 4 °C. After washing in PBST, sections were coverslipped with mounting media (VECTASHIELD Antifade Mounting Medium with DAPI, Vector Laboratories). The average numbers of cells visualized from three adjacent regions at the core/penumbra junction were recorded for each mouse and quantification was done using Fiji software. For the quantification of total vessel length Angio Tool was used. Percentage pericyte coverage was estimated by the area of PDGFR-β divided by the lectin area.

### Statistical analysis

Data are presented as mean ± SEM except for NDS, which was presented as median (interquartile range) and analyzed with the Mann–Whitney test. The ROUT test was used to analyze outliers. Two-group comparisons were analyzed by unpaired t-test with Welch’s correction. Flow cytometry data were analyzed using two-way ANOVA with Tukey’s multiple comparisons tests. Three-group data were analyzed by ordinary one-way ANOVA with Tukey’s multiple comparisons tests. Statistical significance was set at *p* < 0.05.

## Results

### Brain CD13 expression and CD13^+^ monocytes and neutrophils infiltration increased at acute time points after stroke

Stroke responses are biphasic in nature comprising both acute and chronic mechanisms [41]. To investigate the early changes in brain CD13 expression (corresponding to the peak time of inflammation) we performed western blot and flow cytometry analyses on day 3 post-MCAO (Additional file 1: Fig. S1). Western blot findings showed an increase (*p* < 0.05) in cortical CD13 expression on day 3 post-MCAO (Additional file 1: Fig. S1A) compared to sham. As CD13 plays a role in trans-migration [27, 42], we examined brain CD13^+^Ly6G neutrophils and Ly6c^hi^ inflammatory monocytes, early responders after ischemic stroke. The percentage of infiltrated CD13^+^Ly6G neutrophils and Ly6C^hi^ monocytes was higher in the animals that underwent MCAO suggesting a trans-migratory role of CD13 after stroke (Additional file 1: Fig. S1B).

### CD13^+^ endothelial or mural cells increase in both mouse and human brains after a stroke

CD13 is expressed on peripheral myeloid cells [43, 44] including infiltrated monocytes and neutrophils (Additional file 1: Fig. S1B), however little is known about the expression of CD13 on brain resident cells including astrocytes, microglia, endothelial, and pericytes. Our immunohistochemistry findings revealed that CD13 did not co-localize with GFAP^+^ astrocytes or Iba-1^+^ microglia but with PDGFR-β^+^pericytes and lectin^+^ blood vessels in the naïve mouse brain (Additional file 2: Fig. S2A and B) consistent with findings from others that demonstrated that CD13 is expressed by brain pericytes [45]. Both PDGFR-β^+^ cells and lectin^+^ cells co-localized with CD13.

**Fig. 1:**
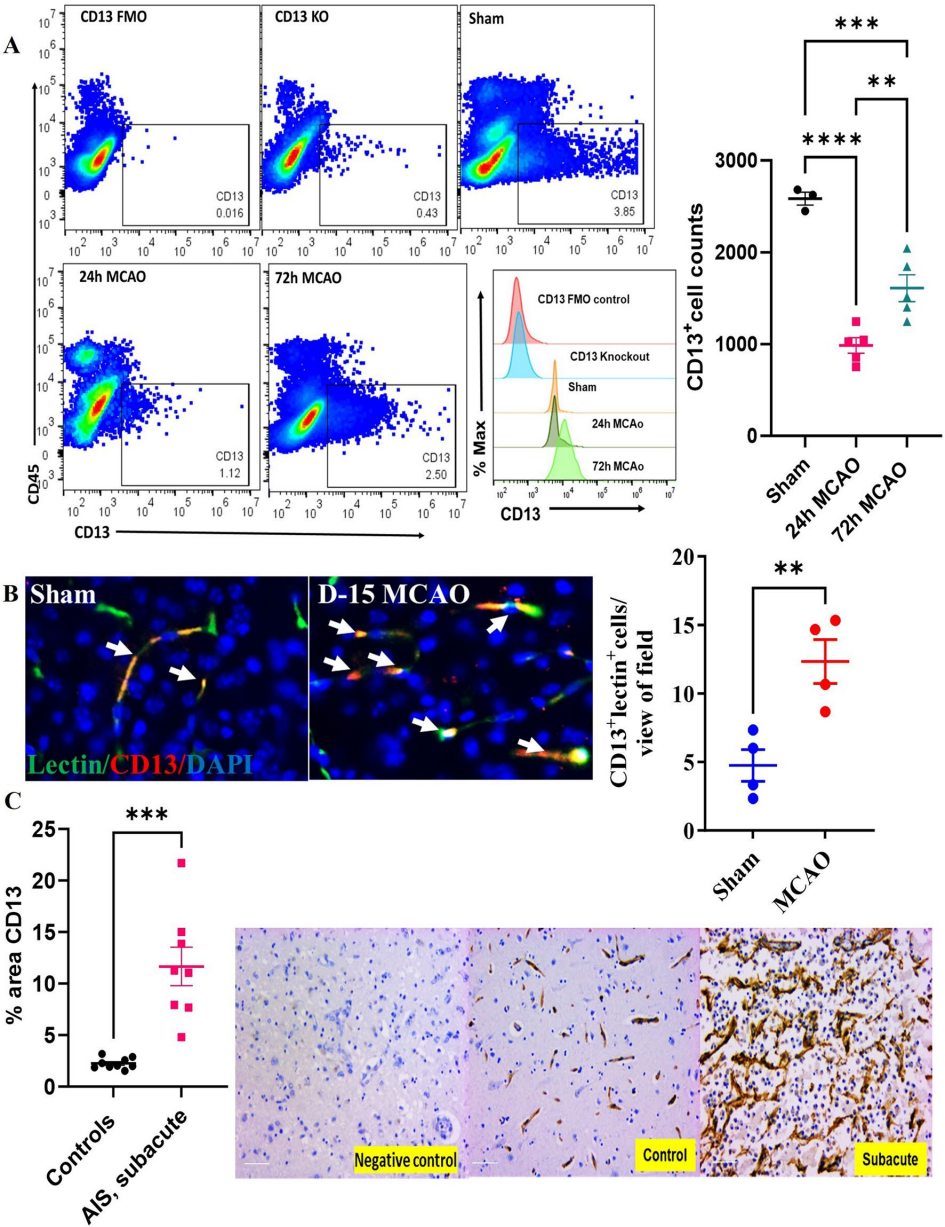
CD13^+^ cells increase in mouse and human brains at a delayed time point after ischemic stroke.** A** A decrease in brain CD13^+^ cell numbers acutely after MCAO and MFI of CD13 after stroke (*n* = 3–5/gp). **B** Increase in the brain CD13^+^lectin^+^cells at day 15 post-MCAO (*n* = 4/gp). **C** An increase in CD13^+^ percentage area at a subacute time point after stroke in human AIS patients. Magnification 20X, scale bar 50 µm, (*n* = 8–9). Data presented as mean ± SEM. Two-group comparisons were analyzed by unpaired t-test with Welch’s correction. Three-group data were analyzed by ordinary one-way ANOVA with Tukey’s multiple comparisons test (**p < 0.01, ***p < 0.001, ****p < 0.0001)

We also assessed brain CD13^+^ cells (endothelial or mural cells) at 24 and 72 h after MCAO in young mice by flow cytometry (Fig. 1A). We observed a significant decline (*p* < 0.05) in CD13^+^ endothelial or mural cell counts at both 24 h and 72 h after MCAO compared to sham. However, an increase (*p* < 0.05) in CD13^+^ endothelial or mural cell numbers was observed in 72 h versus 24 h MCAO group suggesting a recovery in the cells at this time point. CD13 expression increases in activated/angiogenic endothelial cells [30, 31]. After the experimental stroke, an increase in the vascular area is seen in the ischemic core border within 4–7 days [46]. By days 14–28, angiogenesis is observed in the peri-infarct area [47, 48]. On day 15 post-MCAO an increased number of CD13^+^lectin^+^ cells were seen in the peri-infarct area compared to the sham (Fig. 1B). We further investigated CD13^+^ cells in human control and ischemic stroke patients (Fig. 1C). There was a significant increase in the area of cortical CD13^+^ in subacute stroke brains compared to age-matched controls, and the CD13 staining pattern was primarily around blood vessels. These observations suggested CD13^+^cells decline acutely after MCAO in mice. However, an increase in CD13^+^ 15 days after stroke and in subacute stroke human brains suggests that CD13^+^ increases in the later stages of stroke.

### Loss of CD13 did not affect behavioral outcomes in uninjured mice

To determine if loss of CD13 alone affects brain function, we performed cognitive and motor testing on wild-type and global CD13 knockout (KO) mice. Additionally, IHC was performed to investigate markers of inflammation at baseline (GFAP^+^ astrocytes, and Iba-1^+^ microglia) in the cortex and corpus callosum (CC). There was no difference in cerebral blood flow between CD13KO and WT animals (Additional file 3: Fig. S3A). No difference in distance moved on the open field was seen, and KO mice had no apparent motor deficits (Additional file 3: Fig. S3B). There was no difference between latency to escape (Additional file 3: Fig. S3C) and a similar number of incorrect entries (Additional file 3: Fig. S3D) was seen in the Barnes maze reflecting the absence of baseline memory deficits between the two groups. Histological analyses demonstrated no differences in the cortical intensity of GFAP (Additional file 3: Fig. S3E) or Iba-1 (Additional file 3: Fig. S3F) between CD13KO and WT mice. Similarly, no difference in CC GFAP (Additional file 3: Fig. S3H), Iba-1 (Additional file 3: Fig. S3I), or MBP (Additional file 3: Fig. S3J) intensity was seen between the two groups. Although no difference in the cortical PDGFR-β pericytes coverage (Additional file 3: Fig. S3G) was observed in CD13KO versus wild-type mice, CD13KO mice lacked CD13 positivity but still were positive for PDGFR-β (Additional file 3: Fig. S3K). These findings suggested that CD13 deletion does not affect normal histological or brain function in uninjured animals.

### CD13 led to larger infarcts and promoted neutrophil trans-migration

**Fig. 2:**
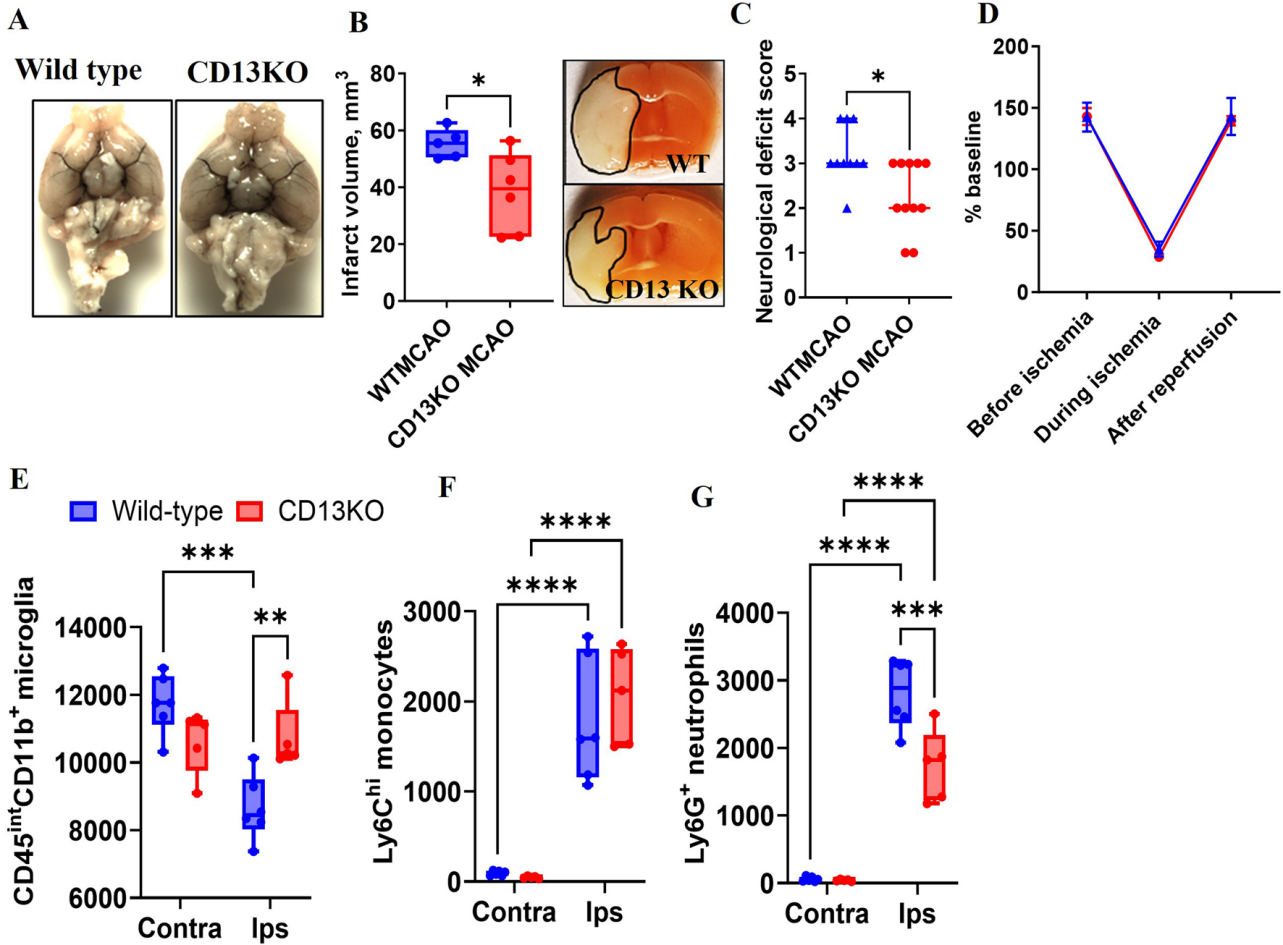
CD13 impedes acute stroke recovery and promotes neutrophil trans-migration. **A** No gross anatomical difference in large blood vessels between wild-type and CD13KO naïve mice (*n* = 3). **B** Smaller infarcts (*n* = 5–6/group) and **C** lower NDS (*n* = 10–11/group) in CD13KO compared to WT after MCAO. **D** No difference in cerebral blood flow between CD13 wild type and CD13 knockout (*n* = 3). **E** Higher microglia counts in the ipsilateral hemisphere of CD13KO mice after stroke. **F** No difference in the infiltrated monocytes in the ipsilateral hemisphere of CD13KO versus WT mice. **G** A decrease in ipsilateral infiltrated neutrophils in the CD13KO mice compared to the WT. *n* = 5–6/gp. Data presented as mean ± SEM. Two-group comparisons were analyzed by unpaired t-test with Welch’s correction. NDS analyzed by Mann–Whitney test and presented as a median with an interquartile range. Two-way ANOVA with Tukey’s multiple comparisons test was used to analyze flow cytometry data (*p < 0.05, **p < 0.01, ***p < 0.001)

To explore the role of CD13 in trans-migration after ischemic stroke, we utilized a global CD13 KO model. The CD13KO naïve mice did not show any gross anatomical difference in large blood vessels of the circle of Willis (Fig. 2A). At 72 h post-MCAO, CD13KO mice had smaller infarcts and improved NDS (*p* < 0.05, Fig. 2B and C) despite a similar drop in CBF (Fig. 2D). These results suggested that CD13 plays a detrimental role in acute stroke pathology and contributes to worsened neurological outcomes.

We then investigated the amount of brain infiltration of myeloid cells in the contralateral and ipsilateral (MCAO side) hemispheres 3 days after stroke. Microglia counts were significantly lower (*p* < 0.05) in the WT ipsilateral compared to the respective contralateral hemisphere, likely representing the increased infarct seen in wild-type mice (Fig. 2E). No difference in microglia numbers in the contralateral and ipsilateral hemispheres was seen in CD13KO mice (Fig. 2E). Microglia numbers were higher (*p* < 0.05) in the CD13KO mice compared to WT ipsilateral hemispheres (Fig. 2E). An increase in ipsilateral Ly6C^hi^ monocytes and Ly6G^+^ neutrophils was observed in the WT and CD13KO animals compared to their respective contralateral hemispheres (Fig. 2F). Although no difference in ipsilateral Ly6C^hi^ monocytes in the CD13KO and WT was observed (Fig. 2F). Furthermore, an increase in ipsilateral Ly6G^+^ neutrophils was observed in both CD13KO and WT compared to their respective contralateral hemispheres (Fig. 2G). However, a significantly lower number of ipsilateral Ly6G^+^neutrophils was observed in CD13KO mice when compared to the WTMCAO mice (Fig. 2G). These results suggested that the reduction in infiltrated neutrophils and higher microglia counts could be partly responsible for the improved stroke outcomes seen in CD13KO mice.

### CD13 plays a critical role in post-stroke cognitive recovery at chronic time points

**Fig. 3:**
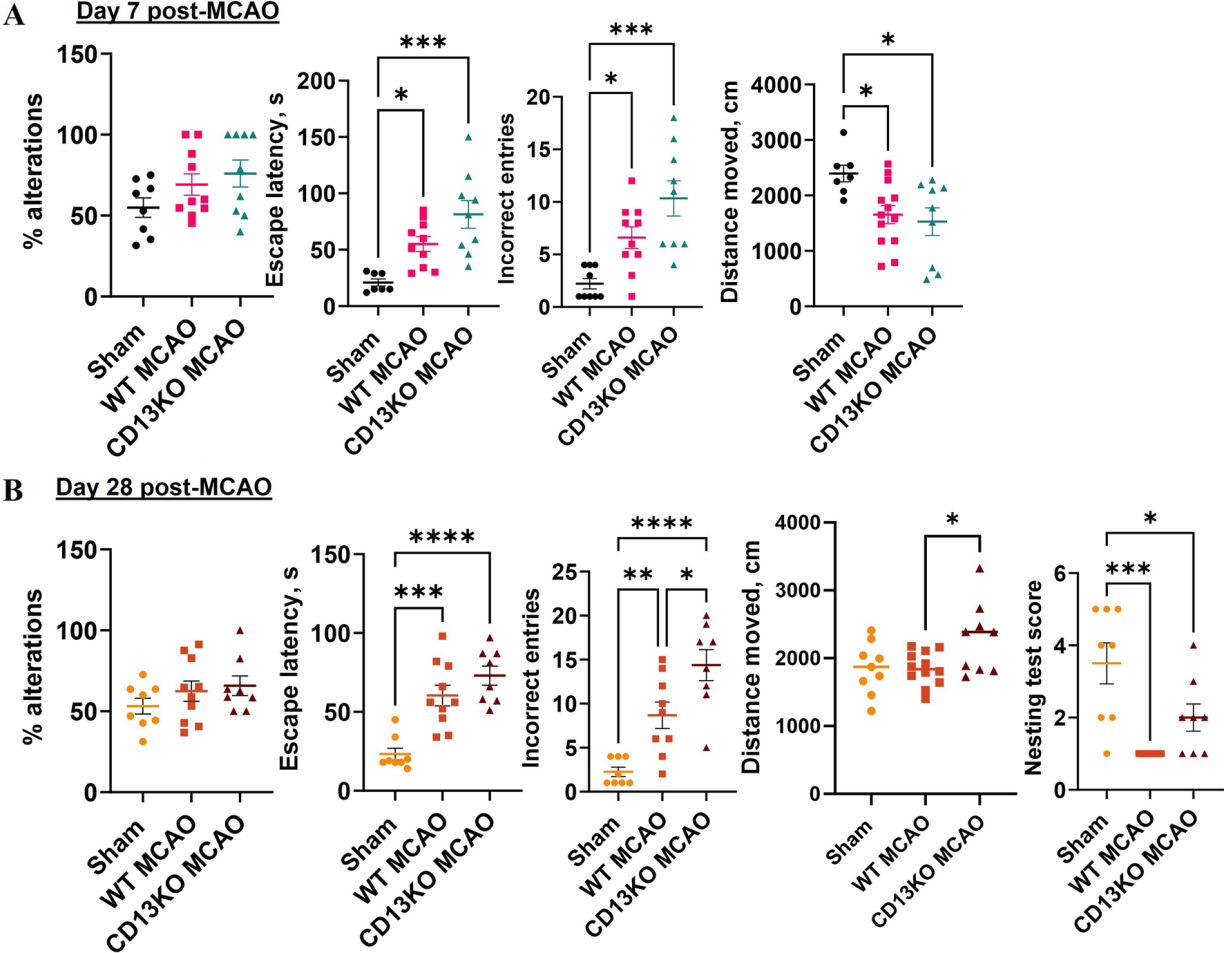
CD13 plays a critical role in post-stroke cognitive recovery at chronic time points. **A** percentage alterations of Y-maze, escape latency, and the number of incorrect entries on Barnes maze, distance moved on an open field at day 7 post-MCAO. **B** percentage alterations of Y-maze, escape latency and the number of incorrect entries on Barnes maze, distance moved on an open field at day 28 post-MCAO. Data presented as mean ± SEM. Three-group data were analyzed by ordinary one-way ANOVA with Tukey’s multiple comparisons test. *n* = 6–13/gp. (*p < 0.05, **p < 0.01)

We investigated functional deficits in WT and CD13KO mice at subacute (Day 7, Fig. 3A), and chronic/recovery (Day 28, Fig. 3B) time points after stroke. On day 7 post-MCAO (Fig. 3A), no difference in percentage alterations on the Y-maze was observed between sham and WTMCAO or sham and CD13KOMCAO animals (Fig. 3A). However, on the Barnes maze, an increase (*p* < 0.05) in the escape latency and incorrect entries was observed between sham and WTMCAO mice. Similarly, CD13KOMCAO mice took a longer time to escape and made more incorrect entries than the sham animals reflecting stroke-induced memory impairment. Although non-significant CD13KOMCAO mice took a longer time to escape and made more incorrect entries than WTMCAO mice. Stroke-induced locomotor activity decreased in both WTMCAO and CD13KO mice compared to the sham at day 7 post-MCAO.

On day 28 post-MCAO (Fig. 3B), no difference in percentage alteration or escape latency was seen between the two MCAO groups however, CD13KOMCAO mice made more incorrect entries on the Barnes maze than the wild-type MCAO mice suggesting a memory deficit. Additionally, the nest-building score was significantly lower (*p* < 0.05) in the WTMCAO mice compared to sham. Similarly, the CD13KOMCAO mice had lower nest-building scores than the shams reflecting to memory impairment [37]. The CD13 KO mice moved more distance on an open field than the WTMCAO animals. No difference in ipsilateral CBF between CD13KO (275.13 ± 22.41 AU) and WT mice (271 ± 22.94 AU) was observed at day 30 post-MCAO. These results showed that the loss of CD13 results in greater cognitive deficit in the subacute and chronic stroke phase, suggesting a beneficial role of CD13 in the functional recovery from stroke.

### CD13 KO mice had increased tissue loss, inflammation, and white matter injury 30 days after the stroke

**Fig. 4:**
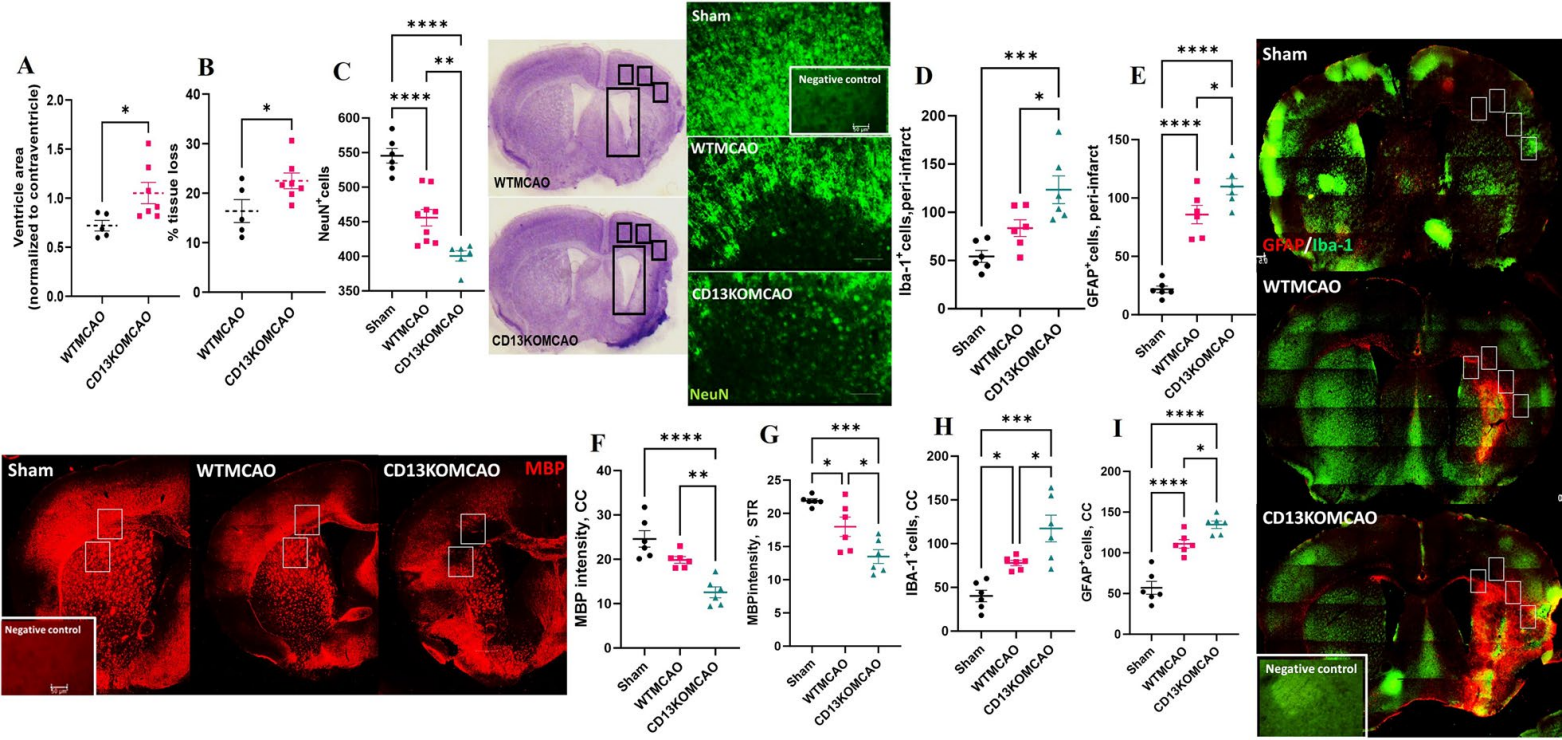
CD13 KO mice had increased tissue loss, inflammation, and white matter injury at day 30 post-MCAO. **A** Ventricle area. **B** percentage tissue loss at day 30 post-MCAO. **C** Neuron counts day 30 post-MCAO. **D** Iba-1 and **E** GFAP counts in the peri-infarct area. **F** MBP intensity in CC and **G** striatum. **H** increased Iba-1 and **I** GFAP counts in CC at day 30 post-MCAO. Magnification 20X. Scale bar 50 µm. Data presented as mean ± SEM. Three-group data were analyzed by ordinary one-way ANOVA with Tukey’s multiple comparisons test. *n* = 6/gp. (*p < 0.05, **p < 0.01, ***p < 0.001)

CD13KO mice had significantly (*p* < 0.05) enlarged ventricles (Fig. 4A) and increased tissue loss (Fig. 4B) compared to WT animals after stroke. MCAO resulted in lower NeuN^+^cells in the peri-infarct region in the WT and CD13KO mice compared to the sham group (Fig. 4C). Lower NeuN^+^ cells were seen in the peri-infarct region (Fig. 4C) in CD13KO animals at day 30 post-MCAO compared to WT mice. Furthermore, MCAO led to markers of inflammation in the peri-infarct region including Iba-1 and GFAP^+^ cells in the peri-infarct area compared to sham (Fig. 4D and E). Iba-1 and GFAP^+^ cells were significantly higher in CD13KOMCAO mice versus WTMCAO animals (Fig. 4D and E). White matter injury (WMI) has been reported after stroke in both clinical and preclinical studies. MBP is a marker for demyelination and is reduced after stroke. A significant (*p* < 0.05) decline in the MBP intensity was observed in the WTMCAO and CD13KOMCAO mice compared to the sham group. A significant (*p* < 0.05) reduction in MBP intensity in the CC and striatum (Fig. 4F and G) of CD13KO as compared to WT mice after stroke, reflecting increased demyelination in the absence of CD13. Increased Iba-1 and GFAP^+^ cells in the CC of CD13KO mice suggested inflammation to be partly responsible for WMI in the CC (Fig. 4H and I).

### CD13 is involved in post-stroke angiogenesis

**Fig. 5:**
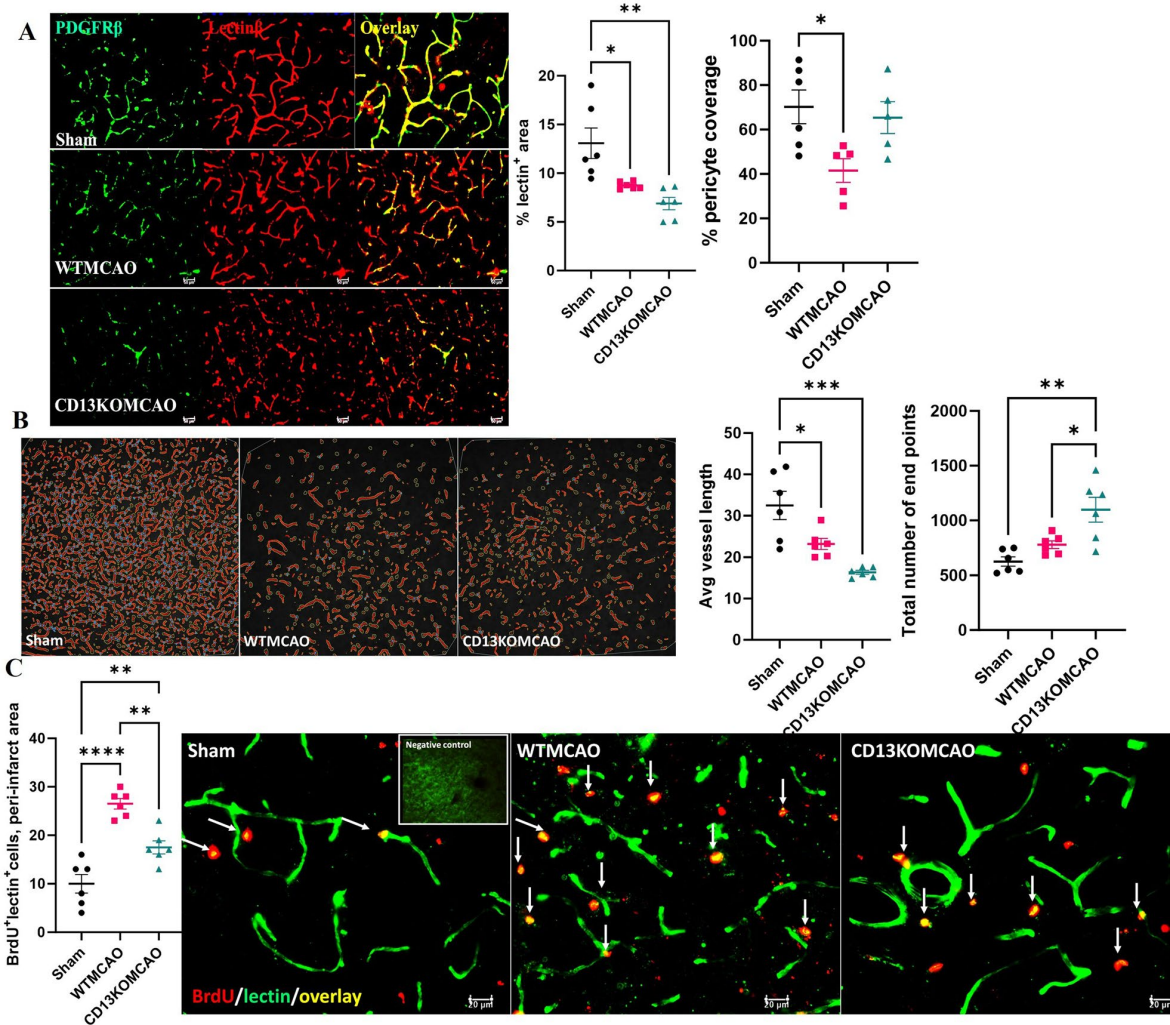
CD13 is involved in post-stroke angiogenesis. **A** Percentage lectin^+^ area and pericyte coverage in the peri-infarct area. **B** Avg. vessel length and the total number of endpoints at day 30 post-MCAO. Magnification 20×. Scale bar 50 µm. **C** BrdU^+^lectin^+^ counts. Magnification 40×. Scale bar 20 µm. Data presented as mean ± SEM. Three-group data were analyzed by ordinary one-way ANOVA with Tukey’s multiple comparisons test. *n* = 6/gp. (*p < 0.05, **p < 0.01)

Angiogenesis and neurogenesis are integral to post-stroke recovery. We used Lycopersicon esculentum (tomato) lectin, DyLight 649 to stain vessels [49]. The percentage lectin^+^ area in the peri-infarct region was significantly reduced in both WT and CD13KO MCAO mice compared to sham (Fig. 5A). PDGFR-β pericyte coverage was significantly reduced in the WTMCAO mice as compared to sham. No difference in PDGFR-β pericyte coverage was observed between CD13KO MCAO and the sham group. Morphometric measurements of the microvasculature were performed with angio Tool. A reduced average vessel length was observed in both WT and CD13KO MCAO mice as compared to sham (Fig. 5B). An increased total number of endpoints were seen in the CD13KO MCAO compared to sham mice (Fig. 5B). CD13KOMCAO mice had an increased total number of endpoints than the WTMCAO mice (Fig. 5B). In addition, higher BrdU^+^lectin^+^ cells were seen in WTMCAO and CD13KOMCAO mice compared to sham (Fig. 5C). CD13KO MCAO mice had significantly lower (*p* < 0.05) BrdU^+^lectin^+^ cells compared to WTMCAO animals suggesting a reduction in angiogenesis (Fig. 5C). To determine the contribution of neurogenesis in post-stroke recovery we examined DCX, a marker for immature neurons in the sub-ventricular zone (SVZ; Additional file 4: Fig. S4A). There was no difference in SVZ DCX^+^ cells between the wild-type and CD13KO MCAO animals (Additional file 4: Fig. S4A). Hippocampal inflammation has been linked to cognitive impairment after stroke [50]. Increased GFAP^+^ and Iba-1^+^ cells were seen in the hippocampus of CD13KO mice (Additional file 4: Fig. S4B) indicative of increased inflammation. These findings implicate CD13 in angiogenesis and hippocampal inflammation.

### Loss of CD13 leads to upregulation of CXCL12 after stroke

**Fig. 6:**
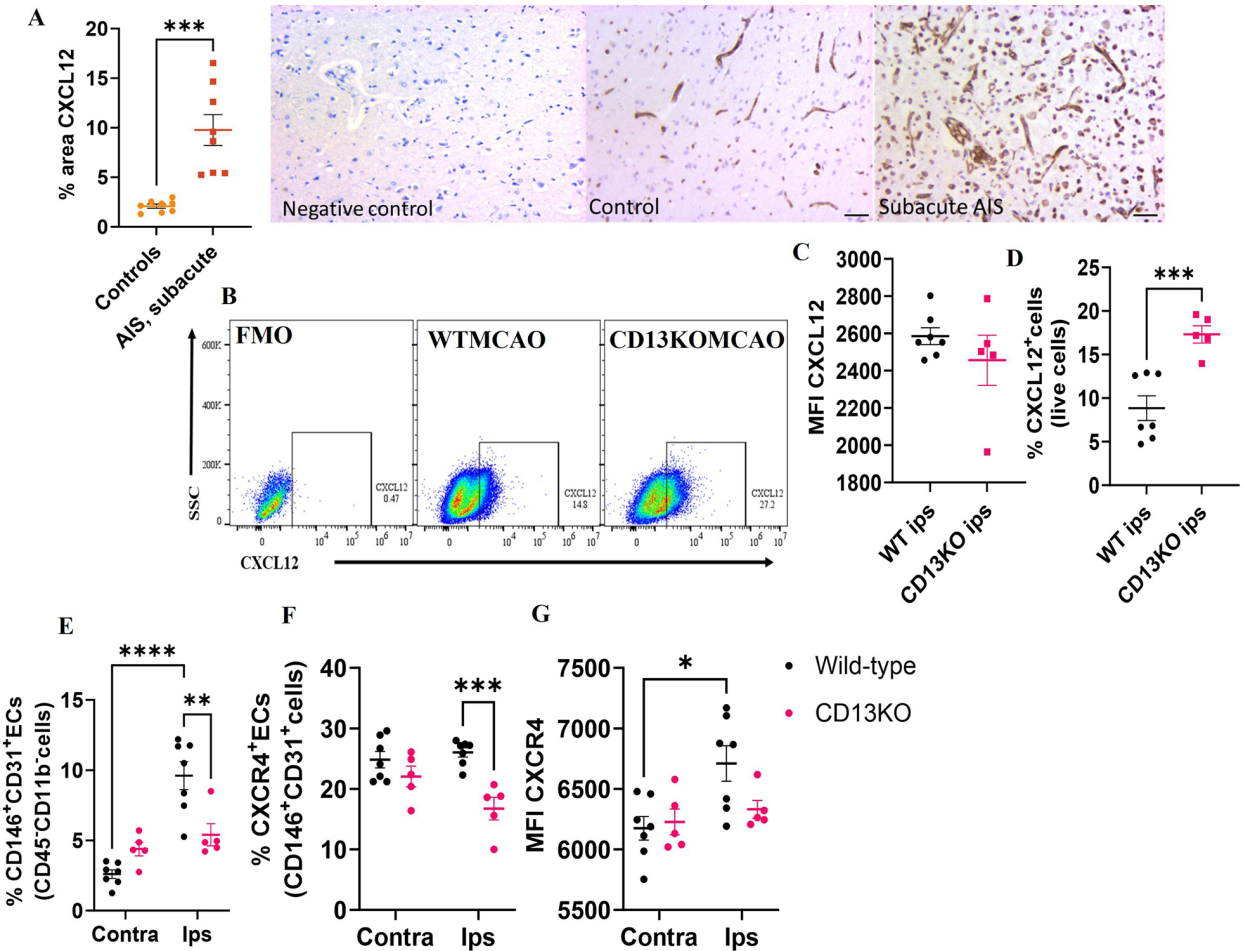
Upregulation of CXCL12/CXCR4 signaling after stroke. **A** Percentage area CXCL12 in human subjects, *n* = 8. **B** Gating strategy for CXCL12 on live cells. **C** MFI CXCL12. **D** percentage CXCL12 on live cells. **E** Ipsilateral and contralateral percentage of CD31^+^ ECs in the wild type and CD13KO mice at day 7 post-MCAO. **F** Percentage of CD31^+^CXCR4^+^ cells. **G** MFI quantification of CXCR4 on CD31^+^ECs. Data presented as mean ± SEM. Data were analyzed using the unpaired t-test with Welch’s correction. Three-group data were analyzed by ordinary one-way ANOVA with Tukey’s multiple comparisons test. *n* = 5–7/gp. (*p < 0.05, **p < 0.01)

CXCL12 is a chemokine involved in inflammation and is secreted by multiple cells after stroke including astrocytes, microglia, endothelial cells, pericytes, and neurons [51]. CXCL12 can play both detrimental and protective roles in stroke [52, 53]. Brain sections from human AIS and age-matched control patients showed that AIS patients had a higher % CXCL12-positive area than age-matched controls at the subacute time point (Fig. 6A). In the mice, we observed no difference in the median fluorescence intensity (MFI) of CXCL12 (Fig. 6C) between the two MCAO groups. However, the percentage of CXCL12^+^ cells significantly increased in CD13KO compared to wild-type animals on day 7 after stroke. This suggests that multiple cell types might be contributing to the stroke-induced increase in CXCL12 (Fig. 6D).

To investigate the role of endothelial cell (EC) CXCL12/CXCR4 pathway after stroke, we then gated on brain ECs as described (Additional file 5: Fig. S5). CD146 is expressed throughout the vasculature and plays a role in cell–cell adhesion, leukocyte trans-migration, and angiogenesis [39, 54, 55]. ECs were gated as CD45^−^CD11b^−^CD146^+^CD31^+^ cells. Stroke induced a greater increase in the % of CD146^+^CD31^+^ ECs seen in the ipsilateral hemisphere of wild-type mice compared to the contralateral hemisphere (Fig. 6E). No difference in % of CD146^+^CD31^+^ ECs was seen in the CD13KO ipsilateral versus the contralateral hemisphere. The percentage of CD146^+^CD31^+^ ECs was significantly lower in the CD13KO mice compared to wild-type animals after stroke (Fig. 6E). CXCL12 signals through CXCR4 and plays a critical role in angiogenesis in experimental intracerebral hemorrhage [56]. In the ipsilateral hemisphere, the percentage of CXCR4^+^ ECs (Fig. 6F) was lower in the CD13KO mice compared to the wild-type after stroke. CXCR4 MFI was increased in the ipsilateral versus the contralateral hemisphere in wild-type MCAO mice (Fig. 6G). A trend toward reduced (*p* = 0.142) ipsilateral CXCR4 MFI was seen in the CD13KO versus wild-type MCAO mice (Fig. 6G). These findings suggested that increased CXCL12 and reduced endothelial cell CXCR4 could be partly responsible for reduced angiogenesis observed in CD13KO mice after stroke.

## Discussion

This work demonstrates several novel findings which support our hypothesis that CD13 contributes towards both acute injury and repair after stroke. Although the loss of CD13 reduced myeloid infiltration and infarct size acutely, later after the stroke, the loss of CD13 was detrimental to recovery. Our findings suggest a biphasic role of CD13 in ischemic stroke pathophysiology. We observed an increase in cortical CD13 expression at day 3 which was consistent with an increase in CD13^+^ monocytes and neutrophils (Additional file 1: Fig. S1) that corroborated with the trans-migratory role of CD13 in the injury [23]. Besides its involvement in cell trafficking, CD13 is also expressed by angiogenic blood vessels and brain pericytes (PCs) [30, 57]. CD13 is used as a sorting marker for pericytes in the mouse brain [58]. CD13 co-localized with PDGFR-β^+^ cells and lectin^+^ cells in the mouse brain and CD13^+^lectin^+^ cell counts were higher at day 15 post-MCAO (Additional file 1: Fig. S1, Additional file 2: Fig. S2). Thus caution should be taken when FACS sorting brain pericytes based on CD13 expression.

After a stroke in both animals and humans, brain PCs and ECs rapidly die [59]. We observed a decrease in brain CD13^+^ cells at both 24 and 72 h after MCAO that could be partially attributed to the loss of PCs and ECs. CD13 is increased in angiogenic blood vessels after ischemic injury in skeletal muscle and cardiac tissue [23, 32]. An augmentation in CD13^+^lectin^+^ cells at 15 days post-MCAO in mice and the increase in CD13^+^ area in stroke patients (Fig. 1) is suggestive of angiogenesis [4, 22].

In healthy mice, CD13 did not influence histological or behavioral outcomes (Additional file 3: Fig. S3), and without injury, CD13 KO mice were indistinguishable from wild-type animals. There was no difference in the anatomy of the vasculature of the circle of Willis, histological markers, or behavior between the two groups, suggesting that CD13 does not contribute to tissue infiltration or functional outcomes in uninjured animals [23, 26, 42]. Furthermore, loss of CD13 did not promote white matter injury [60]. No difference in PDGFR-β pericyte coverage between CD13KO and WT mice was observed. Incidentally, CD13 knockdown did not affect the PDGFR-β counts confirming that CD13 is not exclusive to brain pericytes [58, 61]. After MCAO, a smaller infarct volume, a higher number of surviving microglia, and reduced brain neutrophil infiltration in the CD13KO mice validated the contribution of CD13 in trans-migration during the acute phase of stroke. Interestingly, we did not find a difference in brain monocyte infiltration between the CD13KO MCAO and wild-type MCAO animals suggesting a differential CD13 contribution in monocyte and neutrophil motility. CD13 on activated endothelium interacts with infiltrated monocytes/macrophages and promotes post-stroke angiogenesis and recovery [23, 62]. However, as no difference in the infiltrated monocytes was observed within the two groups, we suspect monocyte/macrophage contribution towards angiogenesis would be similar in the two groups after stroke. Neutrophil CD13 homotypic interactions promote no-reflow [63] and could be in part responsible for increased brain damage in acute ischemic stroke. These observations supported the detrimental role of CD13 in the acute phase of ischemic stroke and that targeting CD13 early could be a viable therapy.

Spontaneous post-stroke functional recovery is seen in young animals after stroke [35, 64]. How CD13 influences long-term functional recovery is unknown. We evaluated motor and cognitive function on day 7 (subacute) and day 28 post (chronic) after the stroke. Both at subacute and chronic time points after stroke, CD13KO animals had worse memory deficits on the Barnes maze suggesting blunted hippocampal-dependent cognitive recovery [65, 66]. Similarly, on day 28 post-MCAO, CD13KO mice were hyperactive in the open field, reflecting persistent habitual impairment [67, 68], or non-associated memory loss [69]. However, no difference in percentage alteration on the Y-maze was observed between the two groups at day 7 or day 28 post-stroke, suggesting that short-term spatial memory [36] was not affected. These observations support the contribution of CD13 in cognitive recovery after stroke.

Both in preclinical and clinical settings, gliosis is a frequent finding that regulates neuroinflammation after stroke [70, 71]. Although CD13 deletion in sham mice did not enhance gliosis, however after stroke CD13KO mice had increased proliferation of Iba-1^+^ microglia and GFAP^+^ astrocytes in the peri-infarct area. Inflammation after stroke causes axonal demyelination resulting in white matter injury, which is associated with long-term cognitive impairment. Increased Iba-1^+^ microglia and GFAP^+^ astrocytes were seen in CD13KO mice after stroke. Increased white matter gliosis may be partly responsible for the reduced corpus callosum and striatum MBP intensity thus culminating in worse cognitive outcomes. Hippocampus inflammation is observed after experimental stroke and is associated with cognitive impairment [50, 72, 73]. The increased hippocampus Iba-1^+^ and GFAP^+^ cells in the CD13KO MCAO mice reflected gliosis and could be partly responsible for dampened cognitive recovery. Overall, these results supported the beneficial role of CD13 in reducing neuroinflammation and ameliorating post-stroke cognitive deficits.

Neurogenesis and angiogenesis promote post-stroke recovery. We did not find a difference in DCX^+^ cells between the wild type and CD13KO MCAO animals suggesting that CD13 is dispensable for post-stroke neurogenesis. PDGFR-β is expressed in neural stem/progenitor cells, and vascular pericytes and is upregulated after cerebral ischemia in both humans and animal models [74, 75]. Additionally, PDGFR-β is shown to be involved in pathological neurogenesis after stroke [76]. No difference in PDGFR-β counts was seen between the wild type and CD13KO MCAO animals thus further supporting that CD13 is unessential in neurogenesis. A decline in the percentage of lectin^+^ area suggested vascular defects in CD13KO MCAO mice supporting the vascular role of CD13. A reduced vessel length and a deficiency in post-stroke angiogenesis in the CD13KO MCAO animals validated the contribution of CD13 in angiogenesis [30, 77]. A reduced lectin^+^ area and BrdU^+^lectin^+^ cells were a consequence of impaired angiogenesis in our study. On the contrary, in the mouse model of myocardial infarction, an increase in CD31^+^ luminal structures in CD13KO mice were seen without improvement in vessel perfusion [23]. Although the authors did not explore the pericyte involvement in their study, they attributed increased angiogenesis to impaired pericyte coverage resulting in uncontrolled endothelial cell proliferation.

Angiogenesis is one of the critical mechanisms for post-stroke recovery. Endothelial migration is essential for angiogenesis. CXCL12 is a chemokine that has been shown to mediate the recruitment and homing of endothelial progenitor cells and promote angiogenesis in an experimental model of myocardial infarction and hind limb ischemia [78–81]. CXCL12 is expressed by multiple cell types including astrocytes, microglia, endothelial cells, and infiltrating immune cells [53, 82, 83], and its expression is elevated in the brain after stroke [84]. Inhibition of CXCL12 reduced peripheral T cell infiltration and improved neurological deficits post-stroke [53]. CXCL12 signals through CXCR4 and CXCR4 are expressed by lymphocytes, endothelial cells, epithelial cells, and stromal cells [51]. BBB-derived CXCL12/CXCR4 signaling promoted NK cell migration and improved functional outcomes after ischemic stroke in mice [52]. In the subacute phase, stroke patients had an increase in CXCL12-positive area compared to controls, reflecting a stroke-induced increase in CXCL12. In experimental stroke, a higher percentage of cells were positive for CXCL12 in the CD13KO MCAO animals. An increased number of activated astrocytes and microglia could be partly responsible for the increased % of CXCL12^+^ cells in the CD13KO MCAO. A decrease in CXCR4^+^ ECs and MFI of CXCR4 suggested impairment in the endothelial CXCL12/CXCR4 pathway which could be partially accountable for reduced angiogenesis in CD13KO MCAO mice. These results support a critical role for CD13 in post-stroke angiogenesis and cognitive recovery.

This is the first study exploring the biphasic contribution of CD13 in stroke. Our study has certain limitations, including the use of a global CD13KO model. Further studies using deletion of myeloid versus endothelial CD13 in inducible models are needed. Stroke is a disease that primarily affects older populations, and we did not assess aged animals. However, our studies validating the involvement of CD13 and CXCL12/CXCR4 in human stroke patients, that were older and both sexes (mean age − 61–92 years) suggests that this is also relevant to clinical stroke. Future studies will require cell-specific studies, pharmacological interventions, and examination of the role of CD13 in both males and females.

In conclusion, CD13 modulates both injury and repair mechanisms. At acute stroke time points, CD13 promotes myeloid cell trans-migration and brain penetration. During the chronic phase, CD13 facilitates angiogenesis and post-stroke recovery.

### Supplementary Information

**Additional file 1: Figure S1.** Brain CD13 expression and infiltrated CD13^+^ monocytes and neutrophils increased at acute time-point after stroke. A. Increase in cortical CD13 expression at 72 h after MCAO. B. Increase in infiltrated CD13^+^Ly6G^+^ neutrophils and Ly6C^hi^ monocytes after MCAO. C. CD13 antibody staining in WT and CD13KO mouse brains. Data presented as mean ± SEM. *n* = 3–5/group. Data were analyzed using the Unpaired t-test with Welch’s correction (*p < 0.05; **p < 0.01).

**Additional file 2: Figure S2. **CD13 co-localizes with PDGFR-β^+^pericytes and lectin^+^ blood vessels in the brain. A. CD13 does not co-localize with Iba-1^+^ microglia and GFAP^+^ astrocytes. B. CD13 co-localizes with PDGFR-β^+^pericytes and lectin^+^ blood vessels in the naïve mouse brain. C. CD13KO mice lack CD13 signal but are positive for Iba-1^+^ microglia and GFAP^+^ astrocytes and D. PDGFR-β^+^pericytes and lectin^+^ blood vessels. E. Negative control. Magnification 40X, scale bar 20 µm. *n* = 3.

**Additional file 3: Figure S3. **CD13 does not affect normal brain functions in young mice. A. Blood flow in the ipsilateral brain hemisphere (*n* = 8–10/gp). B. distance moved on open filed (*n* = 6/gp). C. Escape latency, D. number of incorrect entries on Barnes maze (*n* = 7–9/gp). E. Intensity GFAP. F. Intensity Iba1. G. Percentage pericyte coverage in the cortex (*n* = 3/gp). H. Intensity GFAP. I. Intensity Iba1 and, J. Intensity MBP in CC (*n* = 4–5/gp). K. CD13KO mice lacked CD13^+^ positivity. Magnification 40X, scale bar 20 µm. Data presented as mean ± SEM. Data were analyzed using the Unpaired t-test with Welch’s correction.

**Additional file 4: Figure S4.** No difference in SVZ doublecortin positive cells but increase in hippocampus gliosis in CD13KO mice after stroke A. Doublecortin counts in SVZ. B. GFAP and Iba-1^+^ count in the hippocampus at day 30 post-MCAO. Data presented as mean ± SEM. Magnification 20X. Scale bar 50 µm. Data were analyzed using Ordinary one-way ANOVA with Tukey’s multiple comparisons test. *n* = 6/gp.

**Additional file 5: Figure S5.** Gating strategy for endothelial cells.

**Additional file 6: Figure S6. **Gating strategy for microglia and infiltrated monocytes and neutrophils.

## Data Availability

All the data supporting the findings of this study are available on request from the corresponding author.

## Abbreviations

MCAO: Middle cerebral artery occlusion
CC: Corpus callosum
DCX: Doublecortin
MBP: Myelin basic protein
BBB: Blood–brain barrier
CV: Cresyl violet
KO: Knockout
WMI: White matter injury
NDS: Neurological deficit scores
IHC: Immunohistochemistry
TTC: 2,3,5-Triphenyltetrazolium chloride
AU: Arbitrary units
WT: Wild type
CXCL12: C-X-C motif chemokine 12
CXCR4: C-X-C chemokine receptor type 4
ECs: Endothelial cells
MFI: Median fluorescence intensity
PDGFR-β: Platelet-derived growth factor receptor beta
Iba-1: Ionized calcium-binding adaptor molecule 1
GFAP: Glial fibrillary acidic protein
