# Supplementary figures and images for: CD13 facilitates immune cell migration and aggravates acute injury but promotes chronic post-stroke recovery
Source: J Neuroinflammation. 2023 Oct 10;20:232. doi: 10.1186/s12974-023-02918-3 (PMC10566099; doi:10.1186/s12974-023-02918-3)

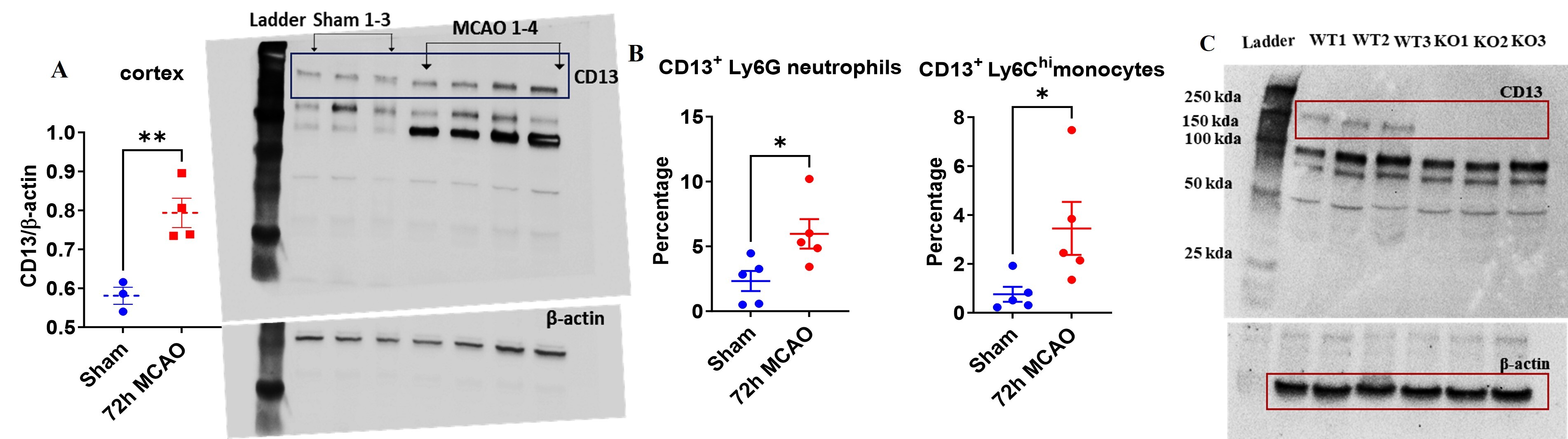

Supplement: Supplementary file 1 — Additional file 1: Figure S1. Brain CD13 expression and infiltrated CD13+ monocytes and neutrophils increased at acute time-point after stroke. A. Increase in cortical CD13 expression at 72 h after MCAO. B. Increase in infiltrated CD13+Ly6G+ neutrophils and Ly6Chi monocytes after MCAO. C. CD13 antibody staining in WT and CD13KO mouse brains. Data presented as mean ± SEM. n = 3–5/group. Data were analyzed using the Unpaired t-test with Welch’s correction (*p < 0.05; **p < 0.01). [file 12974_2023_2918_MOESM1_ESM.jpg]

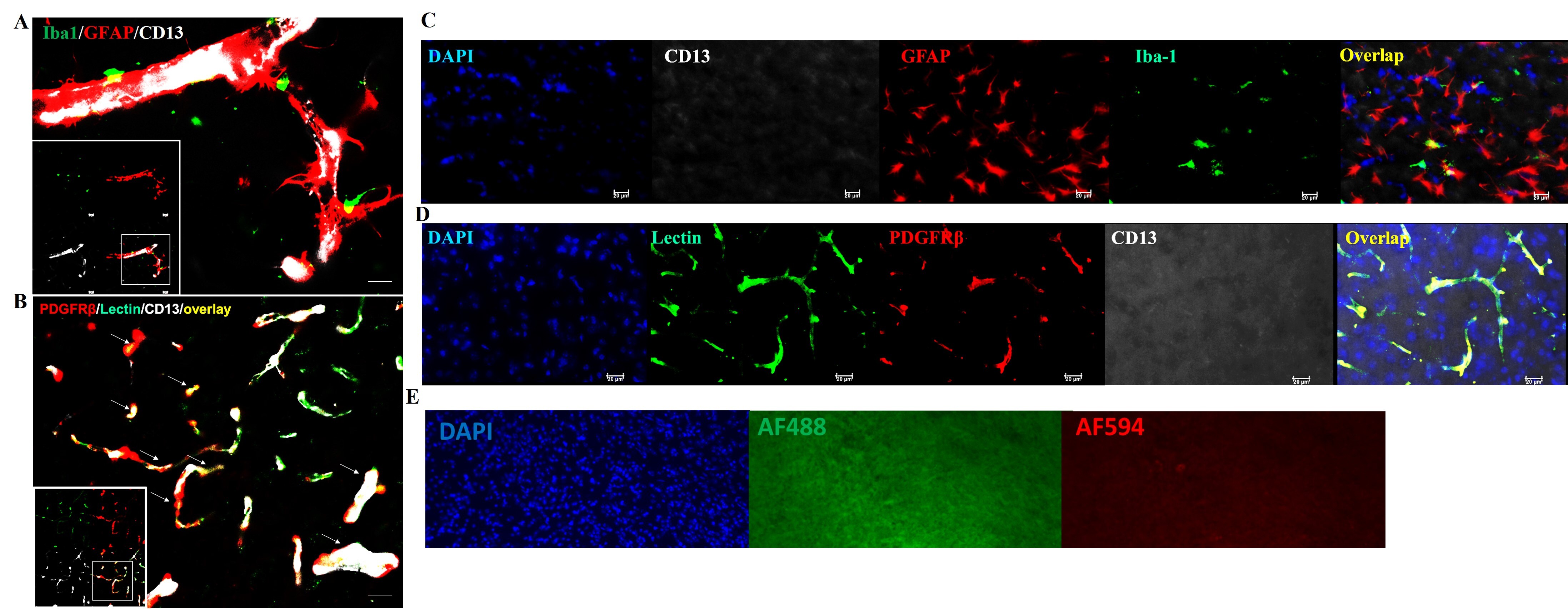

Supplement: Supplementary file 2 — Additional file 2: Figure S2. CD13 co-localizes with PDGFR-β+pericytes and lectin+ blood vessels in the brain. A. CD13 does not co-localize with Iba-1+ microglia and GFAP+ astrocytes. B. CD13 co-localizes with PDGFR-β+pericytes and lectin+ blood vessels in the naïve mouse brain. C. CD13KO mice lack CD13 signal but are positive for Iba-1+ microglia and GFAP+ astrocytes and D. PDGFR-β+pericytes and lectin+ blood vessels. E. Negative control. Magnification 40X, scale bar 20 µm. n = 3. [file 12974_2023_2918_MOESM2_ESM.jpg]

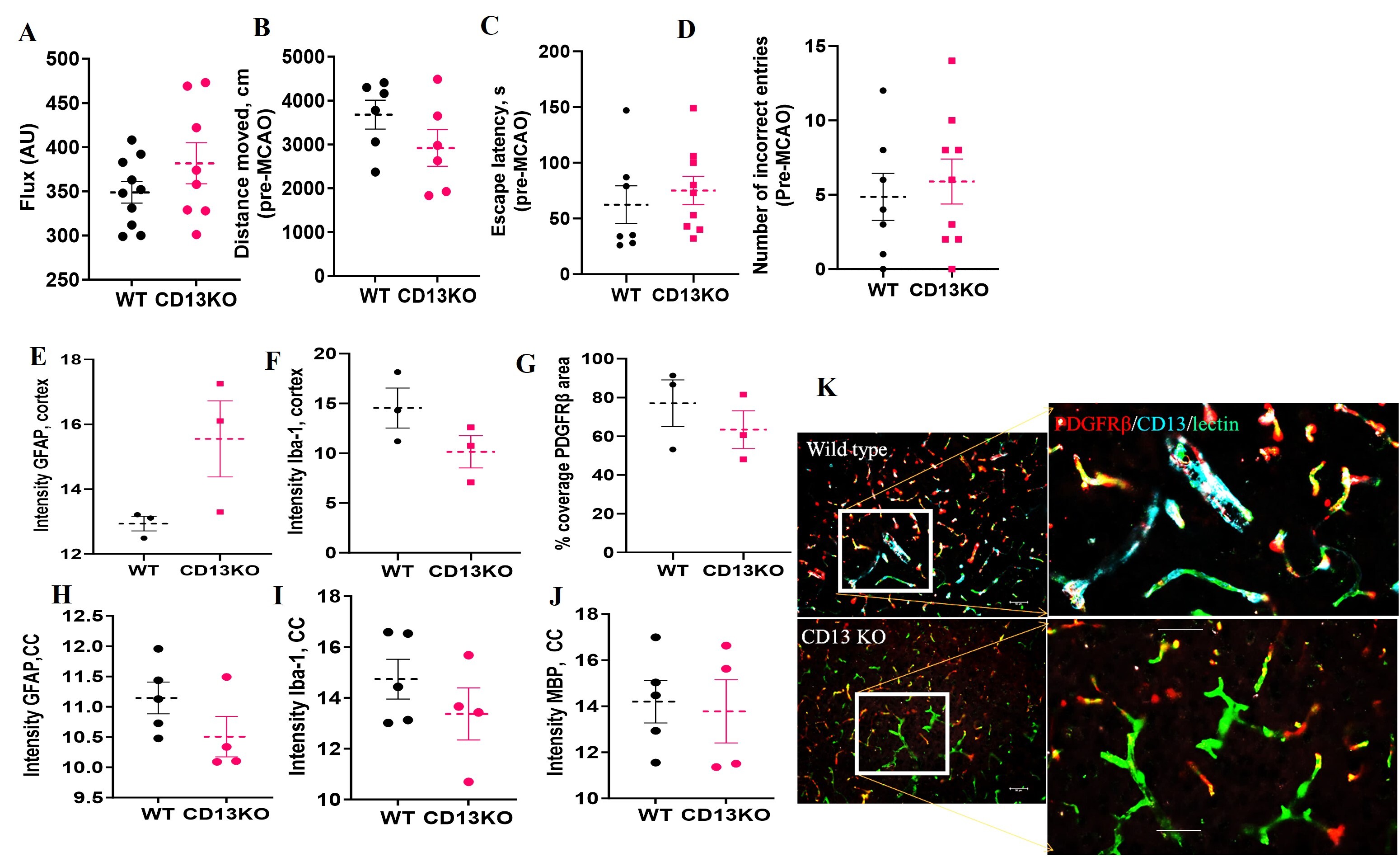

Supplement: Supplementary file 3 — Additional file 3: Figure S3. CD13 does not affect normal brain functions in young mice. A. Blood flow in the ipsilateral brain hemisphere (n = 8–10/gp). B. distance moved on open filed (n = 6/gp). C. Escape latency, D. number of incorrect entries on Barnes maze (n = 7–9/gp). E. Intensity GFAP. F. Intensity Iba1. G. Percentage pericyte coverage in the cortex (n = 3/gp). H. Intensity GFAP. I. Intensity Iba1 and, J. Intensity MBP in CC (n = 4–5/gp). K. CD13KO mice lacked CD13+ positivity. Magnification 40X, scale bar 20 µm. Data presented as mean ± SEM. Data were analyzed using the Unpaired t-test with Welch’s correction. [file 12974_2023_2918_MOESM3_ESM.jpg]

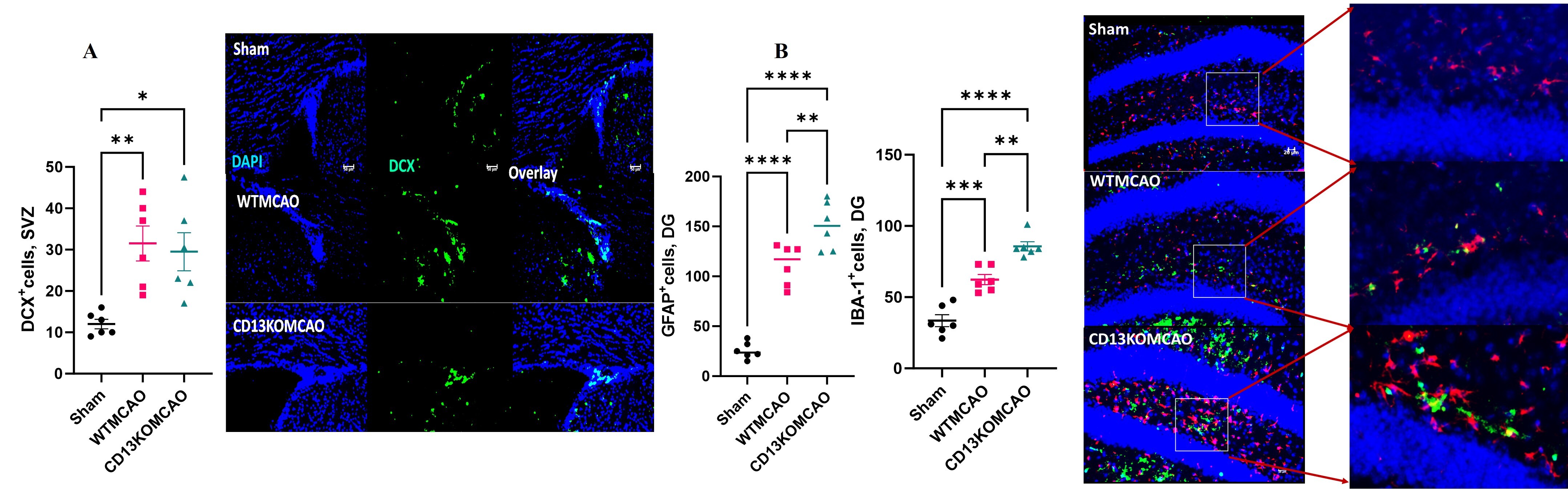

Supplement: Supplementary file 4 — Additional file 4: Figure S4. No difference in SVZ doublecortin positive cells but increase in hippocampus gliosis in CD13KO mice after stroke A. Doublecortin counts in SVZ. B. GFAP and Iba-1+ count in the hippocampus at day 30 post-MCAO. Data presented as mean ± SEM. Magnification 20X. Scale bar 50 µm. Data were analyzed using Ordinary one-way ANOVA with Tukey’s multiple comparisons test. n = 6/gp. [file 12974_2023_2918_MOESM4_ESM.jpg]

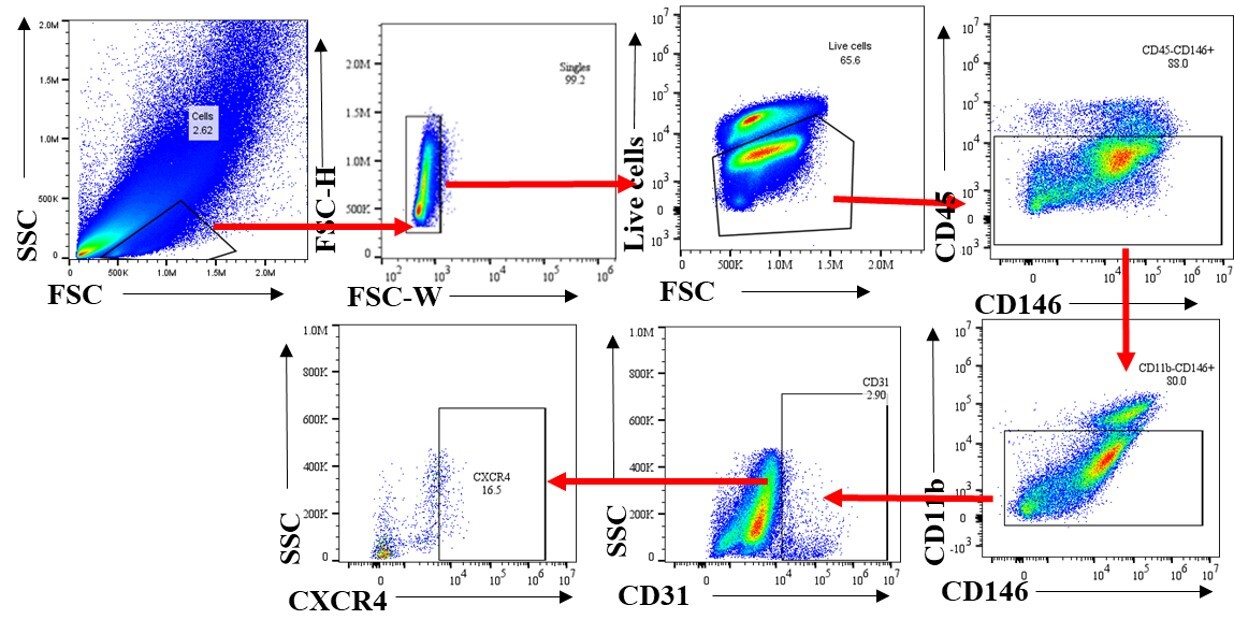

Supplement: Supplementary file 5 — Additional file 5: Figure S5. Gating strategy for endothelial cells. [file 12974_2023_2918_MOESM5_ESM.jpg]

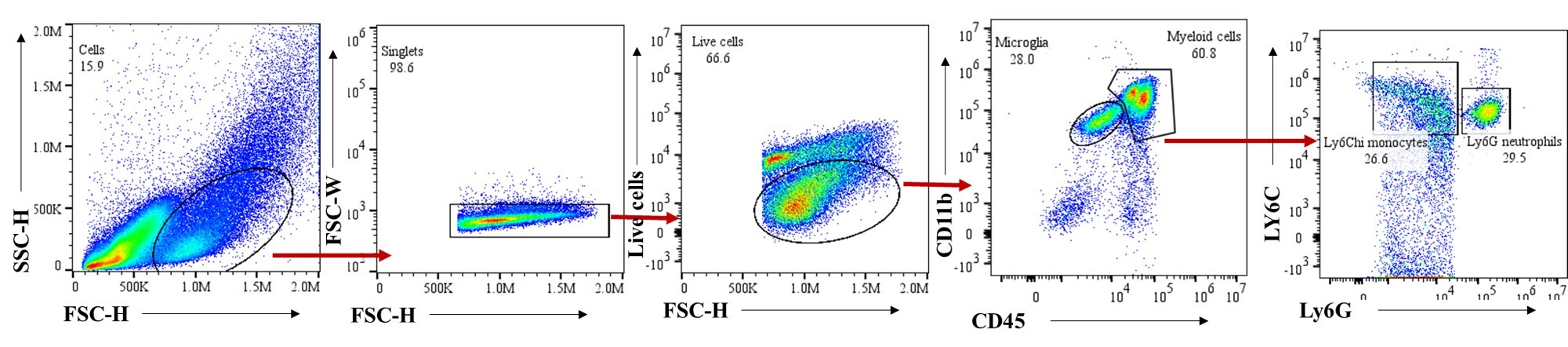

Supplement: Supplementary file 6 — Additional file 6: Figure S6. Gating strategy for microglia and infiltrated monocytes and neutrophils. [file 12974_2023_2918_MOESM6_ESM.jpg]
